# Non-invasive detection of epithelial mesenchymal transition phenotype and metastatic dissemination of lung cancer by liquid biopsy

**DOI:** 10.37349/etat.2021.00032

**Published:** 2021-02-28

**Authors:** Viviana De Rosa, Rosa Fonti, Silvana Del Vecchio, Francesca Iommelli

**Affiliations:** 1 Institute of Biostructures and Bioimaging, National Research Council, 80145 Naples, Italy; 2 Department of Advanced Biomedical Sciences, University “Federico II”, 80131 Naples, Italy; Università degli Studi della Campania “Luigi Vanvitelli”, Italy

**Keywords:** EMT, metastatic dissemination, lung cancer, liquid biopsy

## Abstract

The occurrence of phenotype switch from an epithelial to a mesenchymal cell state during the activation of the epithelial mesenchymal transition (EMT) program in cancer cells has been closely associated with the generation of invasive tumor cells that contribute to metastatic dissemination and treatment failure. Liquid biopsy represents an emergent non-invasive tool that may improve our understanding of the molecular events leading to cancer progression and initiating the metastatic cascade through the dynamic analysis of tumor-derived components isolated from body fluids. The present review will primarily focus on the applications of liquid biopsy in lung cancer patients for identifying EMT signature, elucidating molecular mechanisms underlying the acquisition of an invasive phenotype and detecting new targets for therapy.

## Introduction

**Figure 1.**
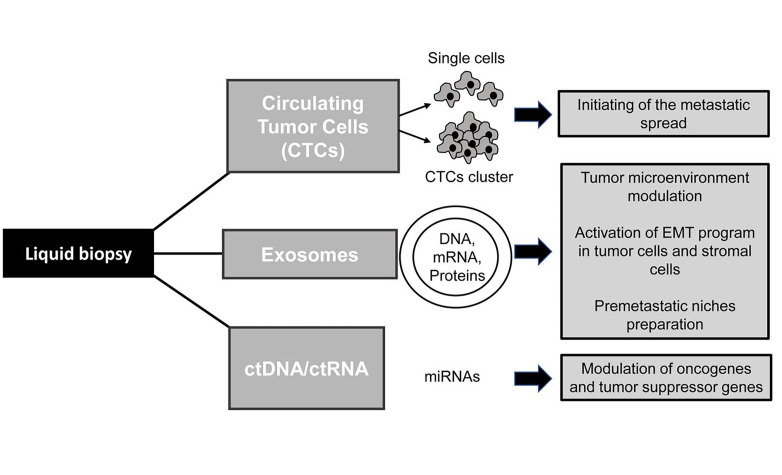
Role of tumor-derived components in cancer invasion

Cancer metastases are the major cause of treatment failure in oncology and many efforts have been made to elucidate the molecular mechanisms causing cancer spread and tumor relapse. In this context, liquid biopsy (LB) represents an emergent non-invasive tool that may be helpful to better understand the biology of invasive carcinomas by the analysis of tumor-derived factors isolated from body fluids. Blood is the most widely used sample for LB but other fluids such as saliva, pleural effusions, urine, or cerebrospinal fluid (CSF) may represent an useful source of cancer biomarkers and the anatomical localization of tumors and their biological properties may affect the presence and types of circulating factors detected in the corresponding body fluids [1, 2]. The clinical application if this innovative approach may be used to collect a plethora of information for improving cancer diagnosis and therapies as for instance the identification of tumor biomarkers, longitudinal monitoring of the drug response and detection of metastasis development at different stages of cancer progression [1]. In particular, LB consists on the identification, and analysis of tumor-derived elements including circulating tumor cells (CTCs), extracellular vesicles (EVs, e.g., exosomes), circulating nucleic acids (ctDNA and ctRNA) and circulating tumor proteins. These elements are released into the blood and other biofluids of the body and may promote tumor growth and progression [3, 4]. Another relevant aspect to consider is that the use of LB in clinical practice may be helpful to overcome the many limitations of conventional tissue biopsy including its invasive nature, patient risk and poor accuracy of the procedure due to the collection of inadequate samples that may not be representative of tumor heterogeneity [2, 5]. However, although some LB-based tests have been approved for cancer patients [1, 6], an accurate detection and characterization of circulating factors remain a challenge in clinical practice and further studies are ongoing to validate the clinical utility of this approach. The present review will primarily focus on the application of LB in lung cancer patients highlighting its role in the detection of the epithelial mesenchymal transition (EMT) and invasive phenotype of CTCs along with the analysis of other tumor-derived components that can modulate cancer microenvironment promoting metastatic spread. Lung cancer is generally diagnosed in its advanced stages and represents one of the leading causes of death worldwide. Histological diagnosis is usually performed by transthoracic or transbronchial biopsy but in some cases, it may be difficult to reach the tumor site and collect the specimen. LB represents an alternative procedure to obtain pathological diagnosis, identify therapeutic targets and detect cancer metastasis in a non-invasive and dynamic manner. The metastatic cascade is a multistep process that starts when tumor cells from the primary site invade the extracellular matrix (ECM), migrate into the circulatory system and reach secondary sites where they promote tumor regrowth [7]. Although several lines of evidence demonstrated that only a small percentage of CTCs is able to survive in the bloodstream and generate metastasis in the body, several studies reported the detection of a high number of CTCs in patients that may indicate the occurrence of metastatic spread and be predictive of a poor prognosis [8–10]. Many efforts to better understand the biology of CTCs and their role in initiating cancer progression are currently ongoing [9, 11]. Several lines of evidences indicate that the EMT, which occurs in a subpopulation of cells within the tumor mass, may be responsible for the generation of CTCs with a high plasticity and migration ability [1, 12]. EMT is a cellular program involved in physiological events such as embryonic development and tissue repair as well as in pathological processes including cancer progression. It is characterized by the cell loss of epithelial traits, disruption of cell junctions and acquisition of an invasive phenotype promoting high cell mobility [13]. Similar to CTCs, other tumor-derived factors may be able to promote cancer cell invasiveness and metastases formation (Figure 1). Notably, exosomes secreted by cancer cells and containing nucleic acids or proteins may trigger the acquisition of a mesenchymal phenotype in malignant cells and mediate the activation of the metastatic cascade [14]. Here we provide representative examples of preclinical and clinical studies describing the utility of LB in advanced lung cancer patients and its ability to detect biological traits of tumor aggressiveness.

## CTCs: detection and characterization

CTCs are a very heterogeneous population of cancer cells detached from primary tumors and/or metastatic lesions and released into the bloodstream during cancer progression. In different tumors CTCs may undergo several phenotypic changes causing resistance to therapies and tumor relapse [15, 16]. The isolation of CTCs from LB is based on their physical and biological properties and may represent an important clinical tool for diagnosis, prognosis, monitoring the response to therapies and improving understanding of tumor progression and metastatic disease [9]. Several studies reported that the identification of high levels of CTCs in blood samples may be predictive of a worse clinical outcome [9, 17]. The predictive value of CTCs number has been also evaluated in lung cancer patients after therapy and it is reported that among treated patients, those who had the highest number of CTCs in the blood, showed a significantly worse prognosis with reduced progression-free survival (PFS) and overall survival (OS). These findings supported the concept that the CTCs number can be modulated by anticancer treatment and used as a surrogate biomarker for tumor response assessment and longitudinal monitoring of disease by multiple liquid biopsies over time [4, 7]. Moreover, *ex vivo* culture of CTCs from patients [18, 19] may be helpful to better understand the CTCs genomic and proteomic profile thus helping the clinicians to personalize the therapy on the basis of the potential targets identified by cell screening. In particular, the detection and classification of cells by biological properties related to the activation of the EMT program (Table 1) may provide information on tumor biology and resistance to treatment on the basis of the enhanced cancer cell migration, high cell plasticity, metabolic reprogramming and stem-like properties detected in CTCs.

**Table 1.** CTCs properties and related markers associated with EMT program in lung cancer

**CTCs properties**	**Biomarkers**	**Ref.**
Epithelial	EpCAM, E-cadherin, CK7, CK8, CK18, CK19	[20, 21]
Mesenchymal	Vimentin, N-cadherin,	[21, 22]
EMT	EpCAM^Low^, E-cadherin^Low^, CK^Low^, Vimentin^High^, N-cadherin^High^, Twist	[21, 22]
Stem-like	CD44, CD133, ALDH1	[17, 22, 23]

EpCAM: epithelial cell adhesion molecule; CK: cytokeratin; ALDH1: aldehyde dehydrogenase 1

### EMT markers

During the last decades, the identification of epithelial markers in CTCs have determined the development of clinically approved methods to detect, isolate and enumerate circulating cells in the blood samples [24]. Although this approach has been widely used in patients, in highly aggressive carcinomas a significant number of CTCs remained undetected due to the total or partial loss of their epithelial signature characterized by the downregulation of proteins such as EpCAM, E-cadherin and CKs [12, 17]. This molecular rearrangement is followed by the acquisition of an EMT phenotype characterized by the upregulation of N-cadherin, fibronectin, vimentin and transcription factors such as Twist, Snail, ZEB and Slug. In this respect, the development of EMT marker-based methods allowed to successfully reveal the presence of CTCs with a mesenchymal signature or with a hybrid phenotype (epithelial/mesenchymal) [25–27]. In agreement with these findings, an interesting study [28] showed high levels of vimentin and low levels of CKs in CTCs derived from metastatic non-small cell lung cancer (NSCLC). In particular, no tumor growth was observed in CDX (patient CTC-derived explant) models after implantation of the CTCs collected before chemo- and radiotherapy whereas CTCs recruited after treatment were able to promote the growth of palpable tumors. Furthermore, the generated CDX were resistant to therapy suggesting the aggressive nature of the injected CTCs. These findings were confirmed by the histopathological analysis of the excised tumors showing poorly differentiated lung adenocarcinoma with a prevalent mesenchymal phenotype as also demonstrated by RNAseq analysis. These evidences shed light on the different biological behaviour between cells from tumors and the tumor-derived circulating cells. In this respect, some authors hypothesized that survival of CTCs in the bloodstream is difficult and therefore it is likely that they undergo a strong selection process leading to survival and proliferation of cells with a more aggressive signature [29]. In agreement with these observations, other studies showed in CTCs, from advanced NSCLC patients, the expression of oncogene drivers such as *EML4-ALK* and mutant *EGFR* associated with the presence of mesenchymal markers [30, 31]. These evidences reinforce the concept that the increase of EMT markers in CTC subpopulations might be a cause of resistance to treatment with tyrosine kinase inhibitors (TKIs) and might be predictive of therapeutic failure. However, despite a large number of studies have been published on this question, the exact role of EMT in resistance to targeted therapies is not been completely elucidated [32].

### EMT activation and stem-like properties in CTCs

Several lines of evidence supported the concept that the activation of an EMT program in CTCs is characterized by a partial and reversible acquisition of a mesenchymal signature [16, 27, 29]. Due to this phenotypic instability, disseminated cancer cells are able to promote adhesion and proliferation at distant sites by restoring their epithelial phenotype. In particular, this plastic and metastable state recognized in CTCs has been similarly observed in cancer stem cells (CSCs) and the activation of EMT in CTCs is indeed associated with the acquisition of stemness properties considered as an additional cause of treatment failure [22, 33, 34]. CSC subpopulations have been isolated both in tumors and in CTCs of several types of carcinomas and have been described as tumor initiating cells with properties of self-renewal, differentiation, inherited drug resistance and high metastatic potential [23]. In CTCs isolated from lung cancer patients, recent studies showed the expression of both mesenchymal and stem cell markers such as OCT4, CD44, CD133, SOX2, ALDH1 [22, 35, 36]. However, despite these observations no statistically significant correlation was found between the activation of EMT and CSC pathways in CTC subpopulations and further studies are needed to better understand the link between the two biological programs that in combination may drive the development of an invasive phenotype and resistance to treatment [33, 36, 37]. In particular, several studies have reported that the activation of EMT programme in cancer cells may determine the acquisition of CSC properties through its effects on both the extracellular and intracellular signaling cascades. However, the contribution of the EMT programme to promote a stem-like phenotype may be variable and most likely depending on cell types and on their genetic or epigenetic properties [33]. Interestingly, stemness markers and properties were also found upregulated in CTCs clusters, which have been identified in the bloodstream as cell aggregates with a more potent ability to form distant metastases than individual circulating cells [38]. The biological process by which CTC clusters originate is still not completely note. The occurring of EMT might induce the expression of the tight junctional proteins enhancing the formation of cell aggregates for collective migration [9, 38] in body fluids. In particular, the access of CTC clusters into the blood stream could be made possible by the porous and leaky blood vessels formed during neo-angiogenesis required for tumor growth. Furthermore, recent studies reported that specific changes in DNA methylation of CTC clusters were associated with the acquisition of a stem-like phenotype and metastases formation [39]. The CD44 receptor seems to have a relevant role in aggregation of CTCs but the exact mechanism of clusters growth and their biology is not completely clear [40, 41]. However, it has been hypothesized that tumor microenvironment and components of ECM may promote the occurrence of this process since a high density of ECM may facilitate tumor cell aggregation and promote cell invasion. Similarly, cytoskeletal and cell adhesion proteins may contribute to CTCs cluster growth and dissemination. Although several techniques are now available to isolate and capture live clusters based on the identification of cell surface markers and size, further efforts are needed to establish the diagnostic and prognostic role of cell aggregates isolated from the bloodstream of cancer patients. In this respect, recent clinical studies have demonstrated a correlation between cluster formation and reduced PFS and/ or OS in different carcinomas including lung cancer [10, 42]. Furthermore, it is reported a lower percentage of apoptotic and proliferating cells within CTCs cluster than in CTC populations thus indicating a major resistance to cytotoxic agents and protection from cell death [43].

### Metabolic program in CTCs

During tumor growth and progression, the activation of EMT program contributes also to modulate the expression of several genes involved in metabolic pathways [44]. Cancer cells adapt their metabolism and energy production to their proliferation program and it is well known that in most tumors less efficient glycolysis is used for the production of ATP and building blocks [45, 46] instead of primarily using oxidative phosphorylation (OXPHOS). However, due to the tumor heterogeneity, several cell subpopulations of the same tumor may exhibit different metabolic phenotypes and interesting studies are ongoing to understand the metabolic signature of CTCs and CTC clusters [38]. Several studies showed that pro-metastatic cells need more glycolytic phenotype for survival. Similarly, Liu et al. [47], reported that subpopulation of stem-like cells isolated from several cancer cell lines, including lung cancer cells, relied more on glycolysis than on OXPHOS. In particular, the authors showed that glucose uptake, several glycolytic enzymes, lactate production, and ATP content were significantly increased in isolated CSC subpopulations. Furthermore, the CSCs fraction was characterized by the suppression of AMPK, activation of Akt pathway and upregulation of pyruvate dehydrogenase kinase (PDK). The increase of PDK levels inhibited the activity of the pyruvate dehydrogenase complex that consequently caused, a suppression of the metabolic flow of pyruvate into the mitochondria, thus promoting the conversion of pyruvate to lactate in the cytosol and the acquisition of a glycolytic phenotype. In another study, Li et al. [48], evaluated the metabolic profile of rare disseminated tumor cells (DTCs) in pleural effusions of 32 lung adenocarcinoma patients in order to reveal metabolic vulnerabilities that could be predictive of therapeutic response. By using a fluorescent glucose analogue 2-NBDG and a mitochondrial redox indicator C12-resazurin (C12R) to probe cellular metabolic activity the authors identified three different metabolically active phenotypes that were independent of oncogenic mutations and were characterized by prevalence of glycolysis, prevalence of OXPHOS or concomitant enhanced glycolysis and OXPHOS. In the same study they calculated a cell number ratio (N/R ratio) that was indicative of the metabolic phenotype and showed that this parameter could be used to predict patient outcome. The three metabolic phenotypes identified by the N/R ratio, were indeed related to the different grade of EMT features and this correlation was used to identify non-responders and short-term beneficiaries among lung cancer patient prior to therapy. Furthermore, it was showed that cells in glycolytic phenotype appeared with an higher mesenchymal signature than cells in mitochondrial oxidation phenotype as confirmed by low expression of epithelial genes and elevated expression of mesenchymal-related genes.

## Tumor-derived EVs

EVs are cell-derived membranous bodies that may be secreted in the biofluids by cells of different tissues acting an autocrine, paracrine or endocrine manner and playing an important role in mediating both physiological and pathological conditions [49, 50]. EVs carry different molecules including DNA, mRNA, proteins and lipids thus inducing modulation of protein expression and functional changes in the recipient cells [51]. Among the different types of isolated EVs, the exosomes have a relevant role in tumor growth and progression and their isolation in patients may represent an innovative tool for diagnosis and therapy in oncology [21, 52]. Exosomes are nanosized vesicles composed of a lipid bilayer that are released from a wide range of cell types such as cancer cells, stroma and endothelial cells [53]. They can transfer information to several districts in the body by multiple mechanisms including fusion with plasma membrane and endocytosis [54]. In particular, recipient cells have been identified in tumor tissue, components of the tumor microenvironment and in distal organs where the exosomes can be delivered by the circulatory system [55]. Recent studies have shown interesting results in lung cancer providing evidence of the role of exosomes in the activation of EMT program and in promoting cancer invasion by modulation of tumor microenvironment and normal cells around the tumor [56]. It has been demonstrated that tumor-derived exosomes, isolated from serum of patients with advanced lung cancer, were able to be captured *in vitro* from normal human bronchial epithelial cells (HBECs) that, upon EVs internalization, showed the acquisition of a metastatic phenotype. These findings were confirmed by the analysis of exosomes contents that showed the presence of both mRNA and proteins associated with mesenchymal properties such as vimentin [53]. Furthermore, it has been reported [57] that tumor-derived exosomes may carry different factors involved in the activation of EMT program including TGFβ, caveolin-1, HIF1α, vimentin and β-catenin thus improving the migration ability of cells inside a tumor and contributing to stromal remodelling. In this respect, it has been hypothesized that tumor associated macrophages (TAMs) and cancer associated fibroblasts (CAFs) may promote cancer cell migration after the internalization of tumor-derived exosomes [58]. Furthermore, nutrient deprivation or low gradient of oxygen may trigger CAFs activation causing the extracellular release of exosomes containing lipids, amino acids and other factors that can induce tumor growth under metabolic stress conditions [59]. Although the exact mechanisms driving the stromal modulation by exosomes have not been completely elucidated, it is hypothesized that this process may be involved also in the preparation of premetastatic niches. In addition, it was reported that hypoxic lung cancer cells can release exosomes that in turn are able to stimulate neo-angiogenesis or create a more favourable tumor microenvironment for metastatic dissemination [60]. In another relevant study [61] exosomes collected from serum of small-cell lung cancer (SCLC) patients showed high levels of FLI1 exonic circular RNA (exo-FECR1) an activator of *FLI1* promoter. FLI1 is aberrantly expressed in TP53 and RB1 deficient SCLC and is associated with late cancer stages and a high proliferation index Ki67. The analysis of exosomes from blood serum samples showed that the levels of circulating exo-FECR1 in patients with advanced metastatic SCLC were higher than in early stage patients and was associated with poor survival and response to chemotherapy. These findings shed light on the potential clinical implications of the isolation and analyse of the circulating exosomes in cancer patients. Although several additional efforts are needed to improve the exosomes identification and extraction from the circulatory system, several techniques are currently available to isolate them from LB such as ultracentrifugation, size exclusion chromatography, density gradients centrifugation and spin column-based methods [55].

## Circulating tumor-derived DNA, RNA and proteins

ctDNA and ctRNA are two other relevant classes of biomarkers identified by LB. They are cell-free circulating nucleic acids derived from the primary tumor, metastatic lesions or CTCs and released in the body fluid mainly after the execution of apoptotic program and cell lysis [5, 62]. High levels of ctDNA and ctRNA have been found in patients with advanced cancer and their characteristics may be related to tumor types and stages [63]. In particular, ctDNA has emerged as a potential tool to identify genetic alterations for targeted therapy and monitor tumor response. In this respect, it has been reported a good concordance rate between activating mutations of EGFR detected in tumor DNA and in ctDNA of NSCLC patients [41, 64, 65]. The identification of mutant ctDNA may also indicate the presence of acquired resistance to EGFR TKIs thus contributing to early detection of treatment failure. Due to this evidence, the first ctDNA-based diagnostic test for *EGFR* mutations has been recently approved by the FDA [66]. Furthermore, recent studies in lung cancer patients reported that the ctDNA analysis may also give an additional contribute in the evaluation of cancer stages and identification of the metastasis sites [67].

More recently, besides ctDNA several studies have also focused on the analysis of ctRNA. In particular, among the different molecules of ctRNAs isolated by LB, microRNA (miRNAs) has emerged as a new tool to better understand tumor biology. miRNAs are short, non-coding single stranded RNA molecules that modulate gene expression by targeting of mRNAs. Circulating miRNAs represent a stable blood-based markers for cancer detection [68] that may be passively released upon cancer cell death and lysis but also actively secreted to mediate intercellular communications. Several miRNAs were isolated from the serum showing ability to modulate the transcription of both oncogenes and suppressor genes [5, 69]. In particular, different exosomal miRNAs have been identified in lung cancer patients and showed a relevant role in tumor growth and progression. They are able to promote different biological processes such as mesenchymal transition, angiogenesis, proliferation and migration [49]. In addition, several studies reported that they may be used as diagnostic and prognostic markers as well as predictive biomarkers for therapeutic response. In this field of study an interesting paper by Cazzoli et al. [70], showed the development of exosomal miRNAs based tests for lung cancer diagnosis. Exososmes were isolated form plasma of patients and RNA extraction were performed followed by quantitative RT-PCR assay. This extensive analysis showed the selection of 6 microRNAs able to discriminate between lung adenocarcinoma and granuloma. In another study, the assessment of exosomal miRNAs levels in plasma were used as a prognostic biomarker of NSCLC patients providing a significant survival prediction [71].

The circulating tumor-derived proteins represent another class of tumor biomarkers widely used in the past years for non-invasive diagnosis, screening and postoperative follow-up of several types of cancer including prostate, colon, breast and lung carcinoma [72]. In particular, the carbohydrate antigen (CA), carcino-embryonic antigen (CEA) and alpha fetal protein (AFP), have been successfully used for advanced lung cancer diagnosis [73]. Furthermore, the assessment of ECM molecules, such as matrix metalloproteinases (MMPs) and fibronectin levels released into the bloodstream from solid tumors, have been identified as promising tool to better understand the tumor microenvironment crosstalk and tumor progression. However, although several other classes of circulating proteins have been detected in cancer patients as potential biomarkers, the approaches to identify individual proteins have generally low sensitivity and specificity and may be associated with high false-positive rate. In this respect, diagnostic platforms using multiple circulating protein markers have been developed to overcome such limitations and improve cancer screening [72].

## Conclusion

Recent advances in the field of tumor biology highlighted the extremely dynamic nature of cancer leading to multiple phenotypic changes occurring during the different phases of cancer progression. The occurrence of these events may result in the acquisition of highly invasive phenotype characterized by the expression of EMT markers and stem-like properties. Due to its minimally invasive nature, LB emerged as an innovative approach that may contribute to the dynamic monitoring of tumor behaviour by multiple analysis of tumor-derived biological components. This approach may be particularly useful in lung cancer which is generally detected at late stages and not easy to be analysed by multiple traditional biopsies. LB may identify very early in the time the initiation of the metastatic process, the activation of EMT program along with the acquisition of a stem-like state in cancer cell subpopulations. However, although some diagnostic tests based on LB have been approved for patients, several additional efforts are needed to improve the efficiency of isolation from body fluids of tumor-derived factors and to achieve standardized protocols for clinical applications.

## Abbreviations

ALDH1: aldehyde dehydrogenase 1
CAFs: cancer associated fibroblasts
CDX: CTC-derived explant
CK: cytokeratin
CSCs: cancer stem cells
CTCs: circulating tumor cells
ECM: extracellular matrix
EMT: epithelial mesenchymal transition
EpCAM: epithelial cell adhesion molecule
EVs: extracellular vesicles
exo-FECR1: FLI1 exonic circular RNA
LB: liquid biopsy
NSCLC: non-small cell lung cancer
OS: overall survival
OXPHOS: oxidative phosphorylation
PDK: pyruvate dehydrogenase kinase
PFS: progression-free survival
SCLC: small-cell lung cancer
TKIs: tyrosine kinase inhibitors

## Declarations

### Copyright

© The Author(s) 2021.

## References

[1] De Rubis G Rajeev Krishnan S Bebawy M. Liquid biopsies in cancer diagnosis, monitoring, and prognosis. Trends Pharmacol Sci. 2019;40:172–86. 10.1016/j.tips.2019.01.006 30736982

[2] Fernández-Lázaro D García Hernández JL García AC Córdova Martínez A Mielgo-Ayuso J Cruz-Hernández JJ. Liquid biopsy as novel tool in precision medicine: origins, properties, identification and clinical perspective of cancer’s biomarkers. Diagnostics (Basel). 2020;10:215. 10.3390/diagnostics10040215 PMC7235853 32294884

[3] Heidrich I Ačkar L Mossahebi Mohammadi P Pantel K. Liquid biopsies: potential and challenges. Int J Cancer. 2020;148:528–45. 10.1002/ijc.33217 32683679

[4] Pantel K Alix-Panabières C. Liquid biopsy and minimal residual disease-latest advances and implications for cure. Nat Rev Clin Oncol. 2019;16:409–24. 10.1038/s41571-019-0187-3 30796368

[5] Rijavec E Coco S Genova C Rossi G Longo L Grossi F. Liquid biopsy in non-small cell lung cancer: highlights and challenges. Cancers (Basel). 2019;12:17. 10.3390/cancers12010017 PMC7017364 31861557

[6] Siravegna G Mussolin B Venesio T Marsoni S Seoane J Dive C How liquid biopsies can change clinical practice in oncology. Ann Oncol. 2019;30:1580–90. 10.1093/annonc/mdz227 31373349

[7] O’Flaherty JD Gray S Richard D Fennell D O’Leary JJ Blackhall FH Circulating tumour cells, their role in metastasis and their clinical utility in lung cancer. Lung Cancer. 2012;76:19–25. 10.1016/j.lungcan.2011.10.018 22209049

[8] Gallo M De Luca A Maiello MR D’Alessio A Esposito C Chicchinelli N Clinical utility of circulating tumor cells in patients with non-small-cell lung cancer. Transl Lung Cancer Res. 2017;6:486–98. 10.21037/tlcr.2017.05.07 28904891 PMC5583074

[9] Dianat-Moghadam H Azizi M Eslami-S Z Cortés-Hernández LE Heidarifard M Nouri M The role of circulating tumor cells in the metastatic cascade: biology, technical challenges, and clinical relevance. Cancers (Basel). 2020;12:867. 10.3390/cancers12040867 PMC7225923 32260071

[10] Kapeleris J Kulasinghe A Warkiani ME Vela I Kenny L O’Byrne K The prognostic role of circulating tumor cells (CTCs) in lung cancer. Front Oncol. 2018;8:311. 10.3389/fonc.2018.00311 30155443 PMC6102369

[11] Lim SB Di Lee W Vasudevan J Lim WT Lim CT. Liquid biopsy: one cell at a time. NPJ Precis Oncol. 2019;3:23. 10.1038/s41698-019-0095-0 31602399 PMC6775080

[12] Jie XX Zhang XY Xu CJ. Epithelial-to-mesenchymal transition, circulating tumor cells and cancer metastasis: mechanisms and clinical applications. Oncotarget. 2017;8:81558–71. 10.18632/oncotarget.18277 29113414 PMC5655309

[13] Nieto MA Huang RY Jackson RA Thiery JP. EMT: 2016. Cell. 2016;166:21–45. 10.1016/j.cell.2016.06.028 27368099

[14] Steinbichler TB Dudás J Riechelmann H Skvortsova II. The role of exosomes in cancer metastasis. Semin Cancer Biol. 2017;44:170–81. 10.1016/j.semcancer.2017.02.006 28215970

[15] Kowalik A Kowalewska M Góźdź S. Current approaches for avoiding the limitations of circulating tumor cells detection methods-implications for diagnosis and treatment of patients with solid tumors. Transl Res. 2017;185:58–84 e15. 10.1016/j.trsl.2017.04.002 28506696

[16] Barriere G Fici P Gallerani G Fabbri F Zoli W Rigaud M. Circulating tumor cells and epithelial, mesenchymal and stemness markers: characterization of cell subpopulations. Ann Transl Med. 2014;2:109. 10.3978/j.issn.2305-5839.2014.10.04 25489583 PMC4245517

[17] Hanssen A Wagner J Gorges TM Taenzer A Uzunoglu FG Driemel C Characterization of different CTC subpopulations in non-small cell lung cancer. Sci Rep. 2016;6:28010. 10.1038/srep28010 27302574 PMC4908396

[18] Maheswaran S Haber DA. *Ex vivo* culture of CTCs: an emerging resource to guide cancer therapy. Cancer Res. 2015;75:2411–5. 10.1158/0008-5472.CAN-15-0145 25998619 PMC4470788

[19] Park SM Wong DJ Ooi CC Kurtz DM Vermesh O Aalipour A Molecular profiling of single circulating tumor cells from lung cancer patients. Proc Natl Acad Sci U S A. 2016;113:E8379–86. 10.1073/pnas.1608461113 27956614 PMC5206556

[20] Genna A Vanwynsberghe AM Villard AV Pottier C Ancel J Polette M EMT-associated heterogeneity in circulating tumor cells: sticky friends on the road to metastasis. Cancers (Basel). 2020;12:1632. 10.3390/cancers12061632 PMC7352430 32575608

[21] Revelo AE Martin A Velasquez R Kulandaisamy PC Bustamante J Keshishyan S Liquid biopsy for lung cancers: an update on recent developments. Ann Transl Med. 2019;7:349. 10.21037/atm.2019.03.28 31516895 PMC6712255

[22] Agnoletto C Corrà F Minotti L Baldassari F Crudele F Cook WJJ Heterogeneity in circulating tumor cells: the relevance of the stem-cell subset. Cancers (Basel). 2019;11:483. 10.3390/cancers11040483 PMC6521045 30959764

[23] Mirza S Jain N Rawal R. Evidence for circulating cancer stem-like cells and epithelial-mesenchymal transition phenotype in the pleurospheres derived from lung adenocarcinoma using liquid biopsy. Tumour Biol. 2017;39:1010428317695915. 10.1177/1010428317695915 28347243

[24] Habli Z AlChamaa W Saab R Kadara H Khraiche ML. Circulating tumor cell detection technologies and clinical utility: challenges and opportunities. Cancers (Basel). 2020;12:1930. 10.3390/cancers12071930 PMC7409125 32708837

[25] Ferreira MM Ramani VC Jeffrey SS. Circulating tumor cell technologies. Mol Oncol. 2016;10:374–94. 10.1016/j.molonc.2016.01.007 26897752 PMC5528969

[26] Wu S Liu S Liu Z Huang J Pu X Li J Classification of circulating tumor cells by epithelial-mesenchymal transition markers. PLoS One. 2015;10:e0123976. 10.1371/journal.pone.0123976 25909322 PMC4409386

[27] Lecharpentier A Vielh P Perez-Moreno P Planchard D Soria JC Farace F. Detection of circulating tumour cells with a hybrid (epithelial/mesenchymal) phenotype in patients with metastatic non-small cell lung cancer. Br J Cancer. 2011;105:1338–41. 10.1038/bjc.2011.405 21970878 PMC3241564

[28] Morrow CJ Trapani F Metcalf RL Bertolini G Hodgkinson CL Khandelwal G Tumourigenic non-small-cell lung cancer mesenchymal circulating tumour cells: a clinical case study. Ann Oncol. 2016;27:1155–60. 10.1093/annonc/mdw122 27013395 PMC4880063

[29] Alix-Panabières C Pantel K. Clinical applications of circulating tumor cells and circulating tumor DNA as liquid biopsy. Cancer Discov. 2016;6:479–91. 10.1158/2159-8290.CD-15-1483 26969689

[30] Pailler E Adam J Barthélémy A Oulhen M Auger N Valent A Detection of circulating tumor cells harboring a unique ALK rearrangement in ALK-positive non-small-cell lung cancer. J Clin Oncol. 2013;31:2273–81. 10.1200/JCO.2012.44.5932 23669222

[31] Lindsay CR Faugeroux V Michiels S Pailler E Facchinetti F Ou D A prospective examination of circulating tumor cell profiles in non-small-cell lung cancer molecular subgroups. Ann Oncol. 2017;28:1523–31. 10.1093/annonc/mdx156 28633480

[32] Zhu X Chen L Liu L Niu X. EMT-mediated acquired EGFR-TKI resistance in NSCLC: mechanisms and strategies. Front Oncol. 2019;9:1044. 10.3389/fonc.2019.01044 31681582 PMC6798878

[33] Shibue T Weinberg RA. EMT, CSCs, and drug resistance: the mechanistic link and clinical implications. Nat Rev Clin Oncol. 2017;14:611–29. 10.1038/nrclinonc.2017.44 28397828 PMC5720366

[34] Mani SA Guo W Liao MJ Eaton EN Ayyanan A Zhou AY The epithelial-mesenchymal transition generates cells with properties of stem cells. Cell. 2008;133:704–15. 10.1016/j.cell.2008.03.027 18485877 PMC2728032

[35] Li S Chen Q Li H Wu Y Feng J Yan Y. Mesenchymal circulating tumor cells (CTCs) and OCT4 mRNA expression in CTCs for prognosis prediction in patients with non-small-cell lung cancer. Clin Transl Oncol. 2017;19:1147–53. 10.1007/s12094-017-1652-z 28374320

[36] Pore M Meijer C de Bock GH Boersma-van Ek W Terstappen LW Groen HJ Cancer stem cells, epithelial to mesenchymal markers, and circulating tumor cells in small cell lung cancer. Clin Lung Cancer. 2016;17:535–42. 10.1016/j.cllc.2016.05.015 27363902

[37] Pirozzi G Tirino V Camerlingo R La Rocca A Martucci N Scognamiglio G Prognostic value of cancer stem cells, epithelial-mesenchymal transition and circulating tumor cells in lung cancer. Oncol Rep. 2013;29:1763–8. 10.3892/or.2013.2294 23426441

[38] Giuliano M Shaikh A Lo HC Arpino G De Placido S Zhang XH Perspective on circulating tumor cell clusters: why it takes a village to metastasize. Cancer Res. 2018;78:845–52. 10.1158/0008-5472.CAN-17-2748 29437766

[39] Gkountela S Castro-Giner F Szczerba BM Vetter M Landin J Scherrer R Circulating tumor cell clustering shapes DNA methylation to enable metastasis seeding. Cell. 2019;176:98–112.e14. 10.1016/j.cell.2018.11.046 30633912 PMC6363966

[40] Amintas S Bedel A Moreau-Gaudry F Boutin J Buscail L Merlio JP Circulating tumor cell clusters: united we stand divided we fall. Int J Mol Sci. 2020;21:2653. 10.3390/ijms21072653 PMC7177734 32290245

[41] Rodrigues P Vanharanta S. Circulating tumor cells: come together, right now, over metastasis. Cancer Discov. 2019;9:22–4. 10.1158/2159-8290.CD-18-1285 30626605 PMC6420083

[42] Balakrishnan A Koppaka D Anand A Deb B Grenci G Viasnoff V Circulating tumor cell cluster phenotype allows monitoring response to treatment and predicts survival. Sci Rep. 2019;9:7933. 10.1038/s41598-019-44404-y 31138856 PMC6538674

[43] Hou JM Krebs MG Lancashire L Sloane R Backen A Swain RK Clinical significance and molecular characteristics of circulating tumor cells and circulating tumor microemboli in patients with small-cell lung cancer. J Clin Oncol. 2012;30:525–32. 10.1200/JCO.2010.33.3716 22253462

[44] Sciacovelli M Frezza C. Metabolic reprogramming and epithelial-to-mesenchymal transition in cancer. FEBS J. 2017;284:3132–44. 10.1111/febs.14090 28444969 PMC6049610

[45] Warburg O. On the origin of cancer cells. Science. 1956;123:309–14. 10.1126/science.123.3191.309 13298683

[46] Vander Heiden MG Cantley LC Thompson CB. Understanding the Warburg effect: the metabolic requirements of cell proliferation. Science. 2009;324:1029–33. 10.1126/science.1160809 19460998 PMC2849637

[47] Liu PP Liao J Tang ZJ Wu WJ Yang J Zeng ZL Metabolic regulation of cancer cell side population by glucose through activation of the Akt pathway. Cell Death Differ. 2014;21:124–35. 10.1038/cdd.2013.131 24096870 PMC3857620

[48] Li Z Wang Z Tang Y Lu X Chen J Dong Y Liquid biopsy-based single-cell metabolic phenotyping of lung cancer patients for informative diagnostics. Nat Commun. 2019;10:3856. 10.1038/s41467-019-11808-3 31451693 PMC6710267

[49] Cui S Cheng Z Qin W Jiang L. Exosomes as a liquid biopsy for lung cancer. Lung Cancer. 2018;116:46–54. 10.1016/j.lungcan.2017.12.012 29413050

[50] Liu S Zhan Y Luo J Feng J Lu J Zheng H Roles of exosomes in the carcinogenesis and clinical therapy of non-small cell lung cancer. Biomed Pharmacother. 2019;111:338–46. 10.1016/j.biopha.2018.12.088 30590322

[51] Lo Cicero A Stahl PD Raposo G. Extracellular vesicles shuffling intercellular messages: for good or for bad. Curr Opin Cell Biol. 2015;35:69–77. 10.1016/j.ceb.2015.04.013 26001269

[52] Zhou B Xu K Zheng X Chen T Wang J Song Y Application of exosomes as liquid biopsy in clinical diagnosis. Signal Transduct Target Ther. 2020;5:144. 10.1038/s41392-020-00258-9 32747657 PMC7400738

[53] Rahman MA Barger JF Lovat F Gao M Otterson GA Nana-Sinkam P. Lung cancer exosomes as drivers of epithelial mesenchymal transition. Oncotarget. 2016;7:54852–66. 10.18632/oncotarget.10243 27363026 PMC5342386

[54] Mathieu M Martin-Jaular L Lavieu G Thery C. Specificities of secretion and uptake of exosomes and other extracellular vesicles for cell-to-cell communication. Nat Cell Biol. 2019;21:9–17. 10.1038/s41556-018-0250-9 30602770

[55] Zheng H Zhan Y Liu S Lu J Luo J Feng J The roles of tumor-derived exosomes in non-small cell lung cancer and their clinical implications. J Exp Clin Cancer Res. 2018;37:226. 10.1186/s13046-018-0901-5 30217217 PMC6137883

[56] Conigliaro A Cicchini C. Exosome-mediated signaling in epithelial to mesenchymal transition and tumor progression. J Clin Med. 2018;8:26. 10.3390/jcm8010026 PMC6352067 30591649

[57] Syn N Wang L Sethi G Thiery JP Goh BC. Exosome-mediated metastasis: from epithelial-mesenchymal transition to escape from immunosurveillance. Trends Pharmacol Sci. 2016;37:606–17. 10.1016/j.tips.2016.04.006 27157716

[58] Fu XT Dai Z Song K Zhang ZJ Zhou ZJ Zhou SL Macrophage-secreted IL-8 induces epithelial-mesenchymal transition in hepatocellular carcinoma cells by activating the JAK2/STAT3/Snail pathway. Int J Oncol. 2015;46:587–96. 10.3892/ijo.2014.2761 25405790

[59] Zhao H Yang L Baddour J Achreja A Bernard V Moss T Tumor microenvironment derived exosomes pleiotropically modulate cancer cell metabolism. Elife. 2016;5:e10250. 10.7554/eLife.10250 26920219 PMC4841778

[60] Hsu YL Hung JY Chang WA Lin YS Pan YC Tsai PH Hypoxic lung cancer-secreted exosomal miR-23a increased angiogenesis and vascular permeability by targeting prolyl hydroxylase and tight junction protein ZO-1. Oncogene. 2017;36:4929–42. 10.1038/onc.2017.105 28436951

[61] Li L Li W Chen N Zhao H Xu G Zhao Y FLI1 exonic circular RNAs as a novel oncogenic driver to promote tumor metastasis in small cell lung cancer. Clin Cancer Res. 2019;25:1302–17. 10.1158/1078-0432.CCR-18-1447 30429198

[62] Hench IB Hench J Tolnay M. Liquid biopsy in clinical management of breast, lung, and colorectal cancer. Front Med (Lausanne). 2018;5:9. 10.3389/fmed.2018.00009 29441349 PMC5797586

[63] van der Vaart M Pretorius PJ. Is the role of circulating DNA as a biomarker of cancer being prematurely overrated? Clin Biochem. 2010;43:26–36. 10.1016/j.clinbiochem.2009.08.027 19747472

[64] Jahangiri L Hurst T. Assessing the concordance of genomic alterations between circulating-free DNA and tumour tissue in cancer patients. Cancers (Basel). 2019;11:1938. 10.3390/cancers11121938 PMC6966532 31817150

[65] Rolfo C Mack PC Scagliotti GV Baas P Barlesi F Bivona TG Liquid biopsy for advanced non-small cell lung cancer (NSCLC): a statement paper from the IASLC. J Thorac Oncol. 2018;13:1248–68. 10.1016/j.jtho.2018.05.030 29885479

[66] Kwapisz D. The first liquid biopsy test approved. Is it a new era of mutation testing for non-small cell lung cancer? Ann Transl Med. 2017;5:46. 10.21037/atm.2017.01.32 28251125 PMC5326656

[67] Zhang L Liang Y Li S Zeng F Meng Y Chen Z The interplay of circulating tumor DNA and chromatin modification, therapeutic resistance, and metastasis. Mol Cancer. 2019;18:36. 10.1186/s12943-019-0989-z 30849971 PMC6408771

[68] Mitchell PS Parkin RK Kroh EM Fritz BR Wyman SK Pogosova-Agadjanyan EL Circulating microRNAs as stable blood-based markers for cancer detection. Proc Natl Acad Sci U S A. 2008;105:10513–8. 10.1073/pnas.0804549105 18663219 PMC2492472

[69] Izzotti A Carozzo S Pulliero A Zhabayeva D Ravetti JL Bersimbaev R. Extracellular microRNA in liquid biopsy: applicability in cancer diagnosis and prevention. Am J Cancer Res. 2016;6:1461–93. 27508091 PMC4969398

[70] Cazzoli R Buttitta F Di Nicola M Malatesta S Marchetti A Rom WN microRNAs derived from circulating exosomes as noninvasive biomarkers for screening and diagnosing lung cancer. J Thorac Oncol. 2013;8:1156–62. 10.1097/JTO.0b013e318299ac32 23945385 PMC4123222

[71] Liu Q Yu Z Yuan S Xie W Li C Hu Z Circulating exosomal microRNAs as prognostic biomarkers for non-small-cell lung cancer. Oncotarget. 2017;8:13048–58. 10.18632/oncotarget.14369 28055956 PMC5355076

[72] Wu J Hu S Zhang L Xin J Sun C Wang L Tumor circulome in the liquid biopsies for cancer diagnosis and prognosis. Theranostics. 2020;10:4544–56. 10.7150/thno.40532 32292514 PMC7150480

[73] Yang B Li X Ren T Yin Y. Autoantibodies as diagnostic biomarkers for lung cancer: a systematic review. Cell Death Discov. 2019;5:126. 10.1038/s41420-019-0207-1 31396403 PMC6683200

